# Supplementary material for: Analytical and clinical validation of direct detection of antimicrobial resistance markers by plasma microbial cell-free DNA sequencing
Source: J Clin Microbiol. 2024 Aug 28;62(10):e00425-24. doi: 10.1128/jcm.00425-24 (PMC11481525; doi:10.1128/jcm.00425-24)
Supplement: Supplemental figures — Figures S1 to S3. [file jcm.00425-24-s0002.pdf]

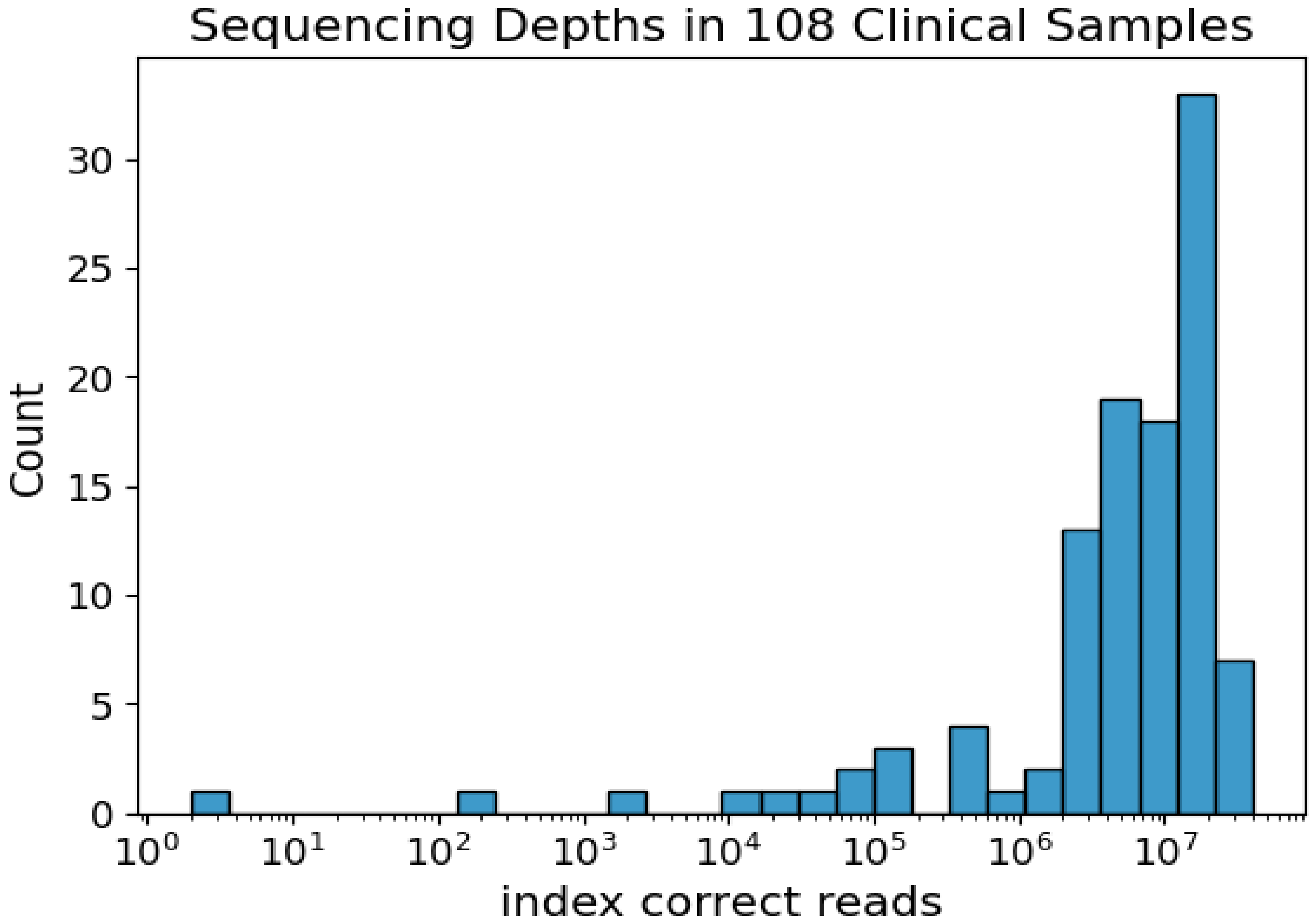

Supplemental Fig. 1. Distribution of index correct read counts across 108 clinical samples.

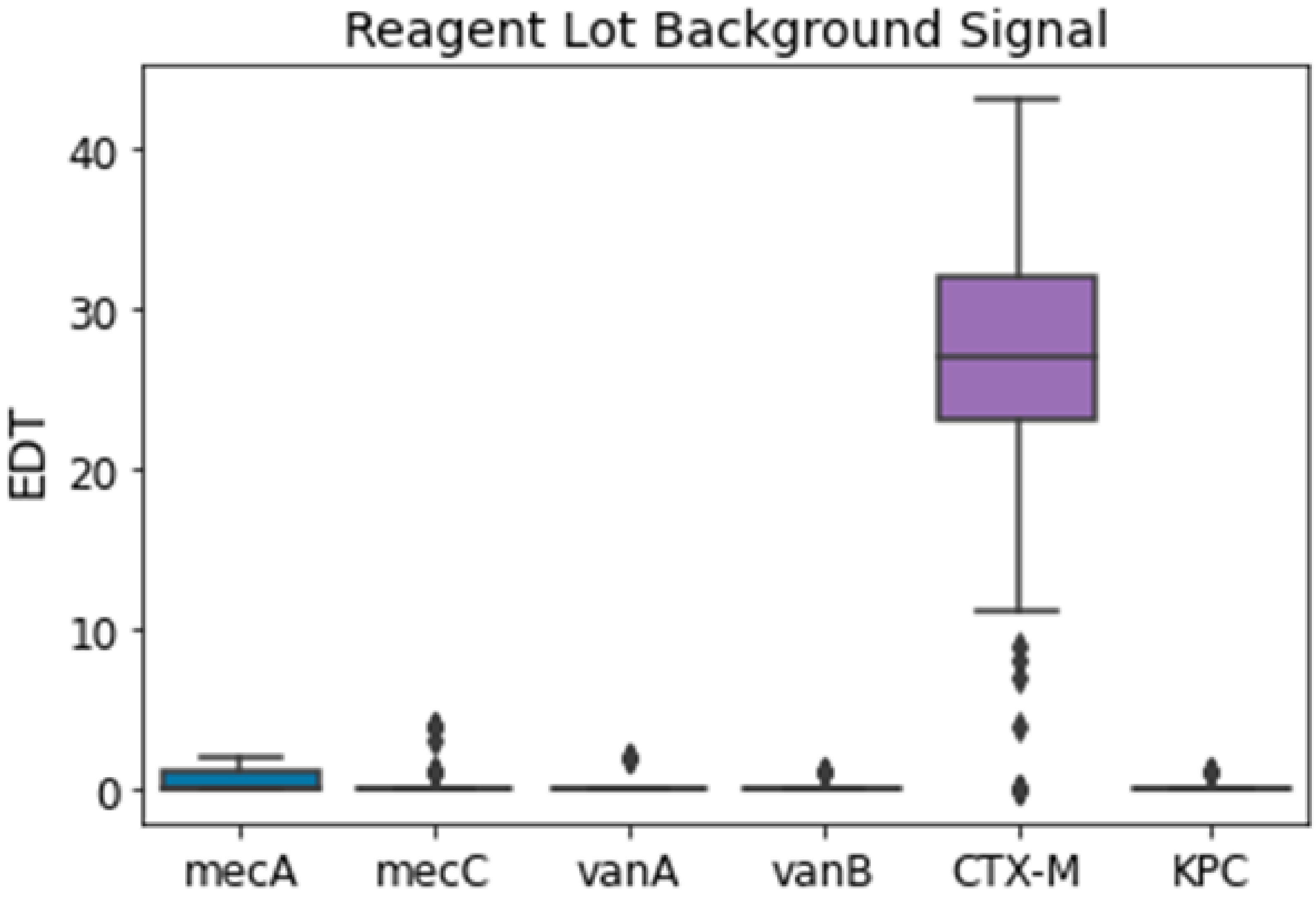

Supplemental Fig. 2. Distribution of EDTs in negative control samples for each AMR target gene.

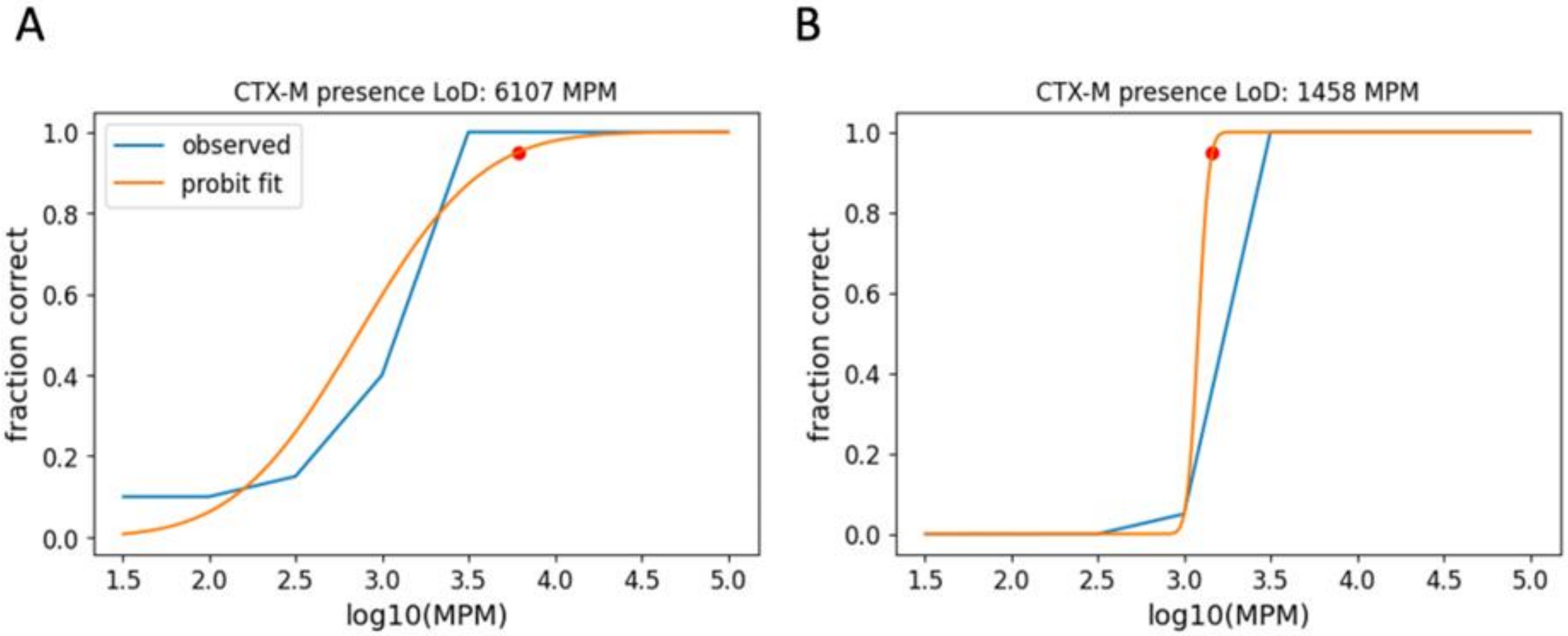

Supplemental Fig. 3. Probit fits for estimating CTX-M presence LoD using the (A) original dilution series and (B) simulated dilution series assuming one CTX-M gene copy. Orange lines reflect the probit model fit and blue lines reflect the observed data of fraction positive calls per concentration.
