# Supplementary material for: Analytical and clinical validation of direct detection of antimicrobial resistance markers by plasma microbial cell-free DNA sequencing
Source: J Clin Microbiol. 2024 Aug 28;62(10):e00425-24. doi: 10.1128/jcm.00425-24 (PMC11481525; doi:10.1128/jcm.00425-24)
Supplement: Supplemental AMR Report — Example of AMR report. [file jcm.00425-24-s0003.pdf]

Sample Karius Test AMR Report and Interpretation.

SPECIMEN: PLASMA

Collected	Nov 27, 2023
Received	Nov 28, 2023
Reported	Nov 29, 2023

PATIENT

Last name	Tester
First name	Patient
Date of birth	Jan 01, 2000
MRN	19023467

ORDERED BY

Clinician Tester
Karius Demo Hospital
975 Island Drive Redwood City CA 94065

TEST RESULTS

MICROBIAL CELL-FREE DNA DETECTED		QUANTITY MOLECULES PER MICROLITER (MPM)[1]	ANNOTATIONS FURTHER INTERPRETATION OF ANTIMICROBIAL RESISTANCE (AMR) ON PAGE 2
**Obligate & Opportunistic Pathogens[2]** Likely to cause disease in humans at any quantity			
Fungi	***Pneumocystis jirovecii*** Alert result	977	
**Commensal Pathogens & DNA Viruses[2]** Known to be associated with disease but may also represent normal microbiota			
Bacteria	***Staphylococcus aureus***	979	• **AMR marker: *mecA* and *mecC* not detected** Consistent with susceptibility to methicillin (methicillin-susceptible *S. aureus*, MSSA).
Viruses	**Cytomegalovirus (CMV)**	1,446	

[1] Molecules Per Microliter = number of DNA fragments present in one microliter of plasma. Visualization of MPM shows quantile of each detected microbe based on 10,000 specimens with positive, quantitative Karius Test results. No quantile is shown if < 20 detections of the microbe were made in the 10,000 specimens or if the microbe is an obligate or opportunistic pathogen. The analytical range of the assay is 10 - 316,000 MPM.

[2] Based on a review of Carroll KC, Pfaller MA. 2019. Manual of Clinical Microbiology, 12th Edition. ASM Press, Washington, DC and Bennett JE, Dolin R, Blaser MJ. 2019. Mandell, Douglas, and Bennett's Principles and Practice of Infectious Diseases, 9th Edition. Elsevier, Philadelphia, PA

Karius staff are available to answer questions about these results at 866-452-7487 or help@kariusdx.com

Test notes

**Nov 30, 2023 (This report)**	Antimicrobial Resistance Addended report released. The test results of this report have been addended with antimicrobial resistance information.
**Nov 29, 2023**	Original report released.

Antimicrobial resistance (AMR) can occur via multiple mechanisms. A "not detected" result for a genetic marker of antimicrobial resistance may not indicate susceptibility to associated antimicrobial drugs or drug classes. A "detected" result for a genetic marker of antimicrobial resistance may not be definitively linked to the microbe(s) detected in some cases. Confirmation of in vitro susceptibility is recommended.

	DETECTED	NOT DETECTED	INDETERMINATE
SCC*mec*, *mecA*, *mecC* [5]	Consistent with resistance to methicillin and all other penicillins, most cephalosporins and carbapenems.	Consistent with susceptibility to methicillin and other β-lactamase-resistant penicillins, cephalosporins and carbapenems.	Antimicrobial susceptibility or resistance of the organism cannot be determined. Causes of an indeterminate result include: insufficient sequence data, ambiguous linkage to specific species due to the presence of confounding microbes, insufficient species-specific markers observed, or ambiguous/ insufficient AMR marker abundance observed.
vanA [6]	Consistent with resistance to vancomycin and other glycopeptides.	Consistent with susceptibility to vancomycin and other glycopeptides.	
vanB [6]	Consistent with resistance to vancomycin.		
CTX-M [7]	Consistent with resistance to extended spectrum and most other cephalosporins, penicillins, and aztreonam.	Unable to determine susceptibility or resistance to extended spectrum cephalosporins and the other drug classes to the left due to multiple other mechanisms of resistance.	
KPC [7]	Consistent with resistance to carbapenems, penicillins, cephalosporins and aztreonam.	Unable to determine susceptibility or resistance to carbapenems and the other drug classes to the left due to multiple other mechanisms of resistance.	

The Karius Test can detect

Full list of microbes: kariusdx.com/karius-test/pathogens

797	206	79	65
Bacteria	Fungi	DNA viruses	Parasites

Test description

The Karius Test for infectious disease detects microbial cell free DNA (cfDNA) in plasma from bacteria, DNA viruses, fungi and protozoa using next-generation sequencing (NGS) [1]. The test reports the presence and abundance of microbial cfDNA when statistically significant levels are detected above background. The test also detects the following antimicrobial resistance (AMR) markers: SCC*mec*, *mecA*, *mecC*, *vanA*, *vanB*, CTX-M,or KPC in relevant microbes (see kariusdx.com/karius-test/amr).

Microbial cfDNA may be found in plasma when viable microbes are not detected in blood by other methods [2]. It can be detected from localized infections or during effective antimicrobial treatment [1, 3, 4]. The reported microbe(s) may or may not be the cause of patient infection. Results should be interpreted within the context of clinical data, including medical history, physical findings, epidemiological factors, and other laboratory data.

Assay limitations

- This test has been validated only for human plasma collected in EDTA anticoagulant.
- Reliable results are dependent on adequate specimen collection, processing, transport, and storage procedures.
- This test has been validated to report only the microbes listed in: kariusdx.com/karius-test/pathogens
- This test detects antimicrobial resistance conferred by the following markers: SCC*mec*, *mecA*, *mecC*,*vanA*, *vanB*, CTX-M, or KPC. Evaluation for these markers will be performed when a microbe known to utilize the antimicrobial resistance mechanism is reported.
  - The antimicrobial resistance marker may not always be linked with the microbe indicated.
  - Presence or absence of an antimicrobial resistance marker does not always correlate to the expected phenotype.
- The assay analytical sensitivity is influenced by the depth of sequencing achieved. A minimum sequencing depth is required to pass quality control. Many batches achieve greater than this minimum sequencing depth resulting in enhanced sensitivity.
- MPM values obtained for different microbes may not be comparable to each other.
- To increase the clarity of the report as it relates to infections, microbes detected as frequently co-occurring are not reported when found together in one specimen. This may reduce the sensitivity to detect polymicrobial events such as mucosal membrane barrier disruptions, skin disruptions, gut injuries or aspiration pneumonia.
- Microbes within a taxonomic family are not reported when detected at less than 25% of the most abundant microbe within the family.
- Microbes within a taxonomic superkingdom are not reported when detected at less than 3% of the most abundant microbe within the superkingdom.
- False positive or false negative results may occur for reasons including but not limited to sporadic contamination from specimen collection, reagent, and materials or hospital and laboratory environments, technical and biological factors.
- The report of a microbe signifies the presence of its cell-free DNA in the patient plasma specimen. It may or may not be the cause of an infection.
- The results obtained from this assay should always be used in combination with clinical examination, patient medical history, and other findings.

Analytical performance and clinical validation

For a summary of the analytical performance and clinical validation see kariusdx.com/karius-test/clinical-and-analytical-validation

MPM interpretation

Positive results will display the concentration of microbial cfDNA detected in units of Molecules of cell-free DNA fragments of a microbe Per Microliter of plasma (abbreviated MPM). The MPM value may be used to infer the amount of microbial cfDNA present in an individual. Several variables impact the MPM value, including the location of infection, prior or ongoing antimicrobial treatment, and genome size of the microbe. In cases where multiple microbes are reported, comparison of MPM values across microbes in the context of etiology should be done with caution.

Antimicrobial Resistance (AMR) testing criteria

AMR testing will be included if the following conditions are met:

- Specimen volume is sufficient for testing
- Microbes associated with the following AMR markers: SCC*mec*, *mecA*, *mecC*, *vanA*, *vanB*, CTX-M, or KPC are detected. See the AMR microbe list at: kariusdx.com/karius-test/amr
- Additional analytical conditions are met

Karius developed this test and determined its performance characteristics

This test has not been cleared or approved by the FDA, nor is it required to be. The Karius laboratory is certified under the Clinical Laboratory Improvement Amendments of 1988 (CLIA '88) and is accredited by the College of American Pathologists (CAP) to perform high-complexity clinical laboratory testing.

Reference interval

The reference interval is derived from a study of 675 asymptomatic adults. Specific reference intervals are calculated using the MPM value reported for the 97.5th percentile for each microbes. For example, the reference interval of E. coli has an MPM value of < 24, which means that across asymptomatic individuals the 97.5th percentile of E. coli quantitations was 24 MPM. MPM values reported below the corresponding reference interval may be the cause of infection, for example due to antibiotic pre-treatment or locus of infection.

Microbes have a reference interval of <10 MPM except for those listed below:

Achromobacter denitrificans < 13
Achromobacter xylosoxidans < 12
Acinetobacter baumannii < 131
Acinetobacter bereziniae < 36
Chryseobacterium indologenes < 182
Enterobacter cloacae complex < 13
Escherichia coli < 24
Haemophilus influenzae < 27
Helicobacter pylori < 78
Klebsiella pneumoniae < 11
Mycobacterium gordonae < 14
Phocaeicola vulgatus (Bacteroides vulgatus) < 13
Pseudomonas aeruginosa < 12
Pseudomonas alcaligenes < 50
Pseudomonas putida < 66
Ralstonia insidiosa < 126
Rhizobium radiobacter (Agrobacterium tumefaciens) < 11
Serratia marcescens < 211
Staphylococcus epidermidis < 17
Stenotrophomonas maltophilia < 127
Streptococcus mitis < 14

References

[1] Blauwkamp T, et al. Nat Microbiol 2019;4(4):663-674.
[2] De Vlaminck I, et al. Cell 2013;155(5):1178-1187.
[3] Farnaes L, et. al. Diagn Microbiol Infect Dis 2019;94(2):188-191.
[4] Rossoff J, et al. Open Forum Infect Dis 2019;6(8).
[5] Lakhundi S, et al. Clin Microbiol Rev 31.4 (2018):e00020-18.
[6] Cetinkaya Y, et al. Clin Microbiol Rev 13.4 (2000):686-707.
[7] Tamma PD, et al. Clin Infect Dis. 2022 Aug 25;75(2):187-212.

Karius, Inc. Laboratory | 975 Island Drive, Ste 101 | Redwood City, CA 94065 | Toll free: (866) 452-7487 | Fax: (866) 246-6567
Email: help@kariusdx.com | CLIA # 05D212136 | CAP # 9497749 | Lab Director: Judith Wilber, PhD, D(ABMM) | v4.2
